# Eukaryotic DING Proteins Are Endogenous: An Immunohistological Study in Mouse Tissues

**DOI:** 10.1371/journal.pone.0009099

**Published:** 2010-02-08

**Authors:** Jean-Marc Collombet, Mikael Elias, Guillaume Gotthard, Elise Four, Frédérique Renault, Aurélie Joffre, Dominique Baubichon, Daniel Rochu, Eric Chabrière

**Affiliations:** 1 Département de Toxicologie, Institut de Recherche Biomédicale des Armées, Centre de Recherche du Service de Santé des Armées, La Tronche, France; 2 Architecture et Fonction des Macromolécules Biologiques, Centre National de la Recherche Scientifique-Aix Marseille Université, Marseille, France; 3 Service de Microscopie et d'Imagerie Médicale, Institut de Recherche Biomédicale des Armées, Centre de Recherche du Service de Santé des Armées, La Tronche, France; Tulane University Health Sciences Center, United States of America

## Abstract

**Background:**

DING proteins encompass an intriguing protein family first characterized by their conserved N-terminal sequence**s**. Some of these proteins seem to have key roles in various human diseases, e.g., rheumatoid arthritis, atherosclerosis, HIV suppression. Although this protein family seems to be ubiquitous in eukaryotes, their genes are consistently lacking from genomic databases. Such a lack has considerably hampered functional studies and has fostered therefore the hypothesis that DING proteins isolated from eukaryotes were in fact prokaryotic contaminants.

**Principal Findings:**

In the framework of our study, we have performed a comprehensive immunological detection of DING proteins in mice. We demonstrate that DING proteins are present in all tissues tested as isoforms of various molecular weights (MWs). Their intracellular localization is tissue-dependant, being exclusively nuclear in neurons, but cytoplasmic and nuclear in other tissues. We also provide evidence that germ-free mouse plasma contains as much DING protein as wild-type.

**Significance:**

Hence, data herein provide a valuable basis for future investigations aimed at eukaryotic DING proteins, revealing that these proteins seem ubiquitous in mouse tissue. Our results strongly suggest that mouse DING proteins are endogenous. Moreover, the determination in this study of the precise cellular localization of DING proteins constitute a precious evidence to understand their molecular involvements in their related human diseases.

## Introduction

DING proteins, named according to their four conserved N-terminal amino-acid residues, encompass a recently discovered protein family [1]. Intriguingly, eukaryotic DING genes are consistently missing from genomic databases although proteins belonging to this family seem to be ubiquitous in eukaryotes: they have been identified in animals (human, monkey, rat, turkey), plants (*Hypericum perforatum*, *Arabidopsis thaliana*, potato, tobacco) and fungi (*Candida albicans, Ganoderma lucidum*) [2], [3] mostly as a 40 kDa protein or higher molecular weight DING proteins [4]. Although no complete eukaryotic DING coding sequence is available in databases, few partial DNA sequences have been either cloned [3] or identified in non-annoted parts of genomes [2]. These few pieces of sequences show that DING proteins are strongly conserved [2]. DING proteins have also been isolated from several prokaryotes [5], [6], and their coding genes are in some cases available, such as the gene encoding PfluDING, the protein isolated from *P. fluorescens* [7], [8], [9]. The high sequence identity between known eukaryotic DING sequences and available prokaryotic DING sequences (>70% sequence identity) and the systematic absence of eukaryotic DING genes raised a controversy about their prokaryotic [10] or eukaryotic origins [11].

DING proteins have been mostly isolated by virtue of a biological function. One of the most striking examples as such, remains the search for a new HIV inhibitor in St John's Wort that led to the characterization of a novel DING protein named p27^sj^ [3]. In humans, several DING proteins have been identified from different tissues [2], [12], [13]. The human phosphate binding protein (HPBP) is a serendipitously discovered plasma apolipoprotein that binds phosphate and is isolated from human plasma [14], [15]. HPBP structure was solved [16] and its physiological function, i.e. its association with paraoxonase (HPON1), an enzyme involved in atherosclerosis [17], has been extensively studied [18], [19], [20]. The complete protein sequence of HPBP was recently determined by a tandem use of mass spectrometry and X-ray crystallography [21]. Such a sequence stands out as the single complete eukaryotic sequence of a DING representative, known to date. Three other human DING proteins involve the crystal adhesion inhibitor (CAI), the human synovial stimulatory protein (SSP), and the X-DING-CD4^+^ from human CD4^+^ T lymphocytes [22]. Comparison of sequenced N-terminal and internal HPBP, CAI SSP, and X-DING-CD4^+^ peptides strongly suggests these stem from 4 different genes, all lacking from the sequenced human genome. The CAI isolated from human kidney cells is assumed to prevent the growth of kidney stones [23]. The SSP, isolated from human synovial liquid, possesses auto-antigen activity, lymphocyte stimulatory activity and a putative role in the etiology of rheumatoid arthritis [24], [25]. The X-DING-CD4^+^ was isolated from CD4+ T cells that are resistant to HIV infection and was shown to block the HIV-1 LTR promoted expression and the replication of HIV-1 [22]. The involvement of DING proteins in a large spectrum of diseases enhances the potential therapeutic value of this specific protein family, and further raises the question of the molecular mechanisms involved in such biological properties.

Although the amount of data concerning this protein family has increased over the last few years, their physiological functions remain largely unknown, and their origin in eukaryotes is still under debate. Hence, we performed a mapping of the DING localization in a murine model.

This study focused on several mouse tissues i.e. brain, liver, lung, heart, aorta, artery and skin. Findings revealed that eukaryotic DING proteins have an intracellular localization. It also provides an essential background for future investigations as regards the numerous biological roles of eukaryotic DING proteins.

## Material and Methods

### Animal Care and Sample Collection

Nine-week old adult male B6D2F1/j@rj mice (Janvier Laboratories - France) were housed in cages under standard conditions of temperature (24°C) and humidity (50–60%), with lights on between 07:00 and 19:00. Mice had free access to water and standard laboratory chow.

All the experiments in our study were reviewed and approved by the Institutional Animal Care and Research Advisory Committee in accordance with French law and leading international guidelines.

Pentobarbital (80 mg/kg) anaesthetized mice were sacrificed by intracardiac perfusion of saline with heparin (5 UI/ml) followed by a fixative solution of 4% formaldehyde and 3% acetic acid in saline. Brain, liver, lung, heart, aorta artery and skin were collected, post-fixed in 4% formaldehyde for 48 hours and then processed for paraffin embedding. For the brain tissue, coronal sections (6 µm thick) were serially cut with a microtome at +0.74 mm (ventricular and striatal areas) and –1.82 mm (hippocampus area) from the bregma, as shown in the Franklin and Paxinos stereotaxic mouse brain atlas [26]. For all other collected tissues (liver, lung, heart, aorta artery and skin), transverse sections were prepared.

Plasma was purified from wild-type and germ-free C57BL/6 mouse blood using standard protocols. Wild-type and germ-free C57BL/6 mouse plasma was kindly provided by the European Mouse Mutant Archive (EMMA; http://emmanet.org).

### Histological Staining

Hemalun-phloxin (H&P) staining was performed on paraffin-embedded tissue sections as previously described by Lillie and Fullmer [27]. For skin and lung sections, a saffron staining step was added after the H&P staining (incubation of sections in 2% saffron solution in ethanol for 10 min).

### Immunohistochemistry

HPBP was purified from human blood plasma as previously described [28]. Rabbit polyclonal and mouse monoclonal antibodies raised against HPBP were prepared by the Genecust Company (Dudelange, Luxembourg) using standard protocols. All polyclonal antibodies and some monoclonal antibodies raised against HPBP are able to recognize other eukaryotic DING proteins, because of the high sequence identity existing in this family.

For DING protein immunohistochemistry, sections from all tested tissues were deparaffinized, rehydrated and treated for 10 min in TBS containing 0.3% v/v H_2_O_2_. For brain tissue, a demasking step in boiling 1 mM EDTA pH 8.0 for 20 min was added. Sections were then successively incubated for 1 h at room temperature in TBS-BSA (TBS containing 1% w/v BSA) and overnight at +4°C in TBS-BSA containing primary antibodies. After 3 washes (15 min each) in TBS, secondary biotinylated IgG antibodies in TBS-BSA were applied to sections for 1 h at room temperature. Visualisation was performed using Vectastain ABC Elite kit (Vector) and diaminobenzidine (DAB; Sigma). Dilutions for primary and secondary antibodies were: as follows 1∶1000 rabbit polyclonal anti-HPBP (antibody C); 1∶50 mouse monoclonal anti-HPBP (antibody 1D3 targeting the N-terminal peptide of HPBP); 1∶400 biotinylated anti-rabbit IgG (Vector) and 1∶1000 biotinylated anti-mouse IgG (Vector). To ensure proper control processing, some sections from all tested tissues were similarly treated except for the fact that the incubation step in monoclonal or polyclonal HPBP primary antibodies was omitted. In addition, to assess the specificity of polyclonal HPBP antibody, a competition experiment was conducted by adding 0.4, 2, 10 or 40 µg of purified HPBP on brain section slides (3 brain sections per slide) in the primary antibody incubation step.

The following markers were considered for visualization of the main types of specific brain cells: GFAP for activated astroglia, *Griffonia simplifolia* lectin (GSA) for activated microglia and NeuN for mature neurons. Double-labeling detections were achieved on deparaffinized and hydrated sections by performing DAB-stained DING protein immunochemistry (brown staining) as described above, followed by GFAP or NeuN immunolabeling using SG vector dye (blue staining) for visualization [29]. For the detection of DING protein in activated microglia, GSA staining was carried out as previously described by Streit [30] with DAB-staining, followed by DING protein immunochemistry using SG vector as dye revelator.

In control mice, GFAP or GSA staining is very faint since activated astroglial and microglial cells are almost completely absent. Therefore, for the specific detection of DING protein in activated glial cells, brain sections from soman-intoxicated mice were used. Soman, an irreversible cholinesterase inhibitor, is a powerful warfare neurotoxicant triggering epileptic seizures leading to neuronal cell death and subsequent glial activation [29]. For this purpose, nine week old adult male B6D2F1/j@rj mice were subcutaneously injected with 110 µg/kg soman (200 µl in saline buffer; soman was provided by the “Centre d'Etudes du Bouchet” - France) followed 1 min. later by an intraperitoneal injection (200 µl in saline) of 5.0 mg/kg atropine methyl nitrate. Pentobarbital (80 mg/kg) anaesthetized mice were sacrificed on day 3 (pick of microglial cell activation) and day 8 (pick of astroglial cell activation) post-poisoning using intracardiac perfusion of formaldehyde and collected brains were processed for immunohistochemistry as described above for control mice.

### Western Blots Assays

Anaesthetized mice were decapitated and tissues such as the whole brain without cerebellum, liver, lung, shaven skin and heart ventricle were immediately collected. Brain and liver were homogenized with a mini-potter in 5 volumes of cold RIPA buffer containing 20 mM Tris-HCl pH 8.0, 150 mM NaCl, 1 mM EDTA, 1% v/v NP40, 0.5% w/v SDS, 0.5% w/v deoxycholic acid and 0.5% v/v protease inhibitor cocktail set III (Calbiochem - Merck). Lung, skin or heart were disrupted using a mixer mill (Retsch MM301) calibrated for two sessions of 2 min shaking each, at 30 Hz. For disruption, tissues were transferred in a 2 ml microfuge tube containing 5 volumes of cold RIPA buffer (see above) and two 3 mm tungsten carbide beads (Retsch). Subsequently, homogenates were centrifuged at 14000 g for 20 min at +4°C and supernatants were frozen at −20°C for further western blot analysis. Prior to freezing, protein concentrations were determined in the supernatants, using the Lowry method [31].

Western immunoblotting was achieved as previously described [32]. For each tissue homogenate or plasma sample, 20 µg of total protein per well were loaded onto a 10% SDS-PAGE gel. Gel electro-transfer was performed onto 0.2 µm nitrocellulose membrane using a specific transfer buffer (48 mM Tris; 39 mM glycine; 20% methanol; 1.3 mM SDS; pH 9.2). For DING proteins detection on tissues, membranes were incubated with 1∶400 monoclonal HPBP antibody and 1∶1000 biotinylated-IgG anti-mouse (Vector). Western blots on plasma samples were performed using 1∶2000 polyclonal anti-DING antibodies and 1∶2500 anti-rabbit antibodies (BioRad). The size of DING proteins bands was calculated according to the migration of Precision plus protein kaleidoscope standard (BioRad) on the same gel for all experiments, except for the western blot on plasma samples, where Prestained Protein Ladder was used (Fermentas life science).

## Results

### Western Blot Analysis

**Figure 1:**
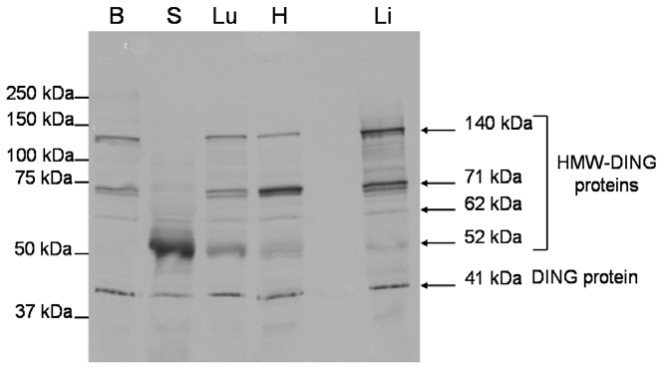
Characterization of DING proteins by western blot analysis. Western blots were achieved with protein homogenates prepared from B6D2F1 mouse brain, shaven skin, lung, heart and liver (loading of 20 µg total proteins in each well). DING proteins were identified with mouse monoclonal anti-DING antibody. Diaminobenzidine was used for the band revelation. Abbreviations are: B  =  brain; S  =  shaven skin; Lu  =  lung; H  =  heart; Li  =  liver; HMW  =  high molecular weight.

Western blot analysis was performed on brain, shaven skin, lung, heart and liver prepared from B6D2F1 mouse samples. Anti-DING monoclonal antibody revealed several bands ranging from 41 to 140 kDa in all tested mouse tissues (**Figure 1**). The 41 kDa protein band corresponds to the size of eukaryotic DING proteins already described in the literature [1]. Intensities of 41 kDa bands were very similar in all tested mouse tissues except in shaven skin where the band intensity is weaker. Other bands between 52 and 140 kDa are more likely high molecular weight DING proteins (HMW-DING) comparable to the ones already observed in plants [4]. A similar band pattern was found in mouse lung, heart and liver with 4 major HMW-DING bands located at 140 kDa, 71 kDa (double band of HMW-DING), 62 kDa and 52 kDa. In the heart, the intensity of the double 71 kDa band is more important than the one detected in lung and liver. In the brain, the 3 HMW-DING bands at 140 kDa, 71 kDa (double band) and 62 kDa are revealed but the 52 kDa HMW-DING protein was not present. In the shaven skin, only one HMW-DING was identified with a very intense band located at 52 kDa (**Figure 1**).

**Figure 2:**
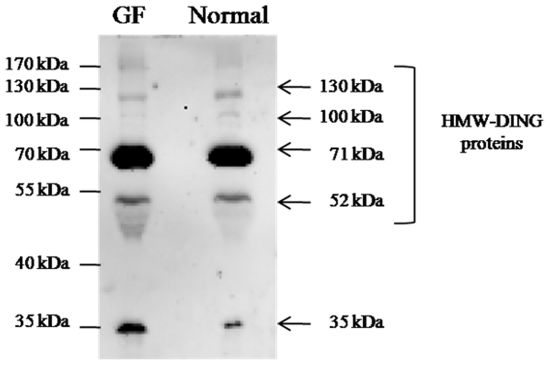
Western blot analysis on plasma samples from normal and germ-free mouse. Western blot was achieved with plasma from wild type and germ-free C57BL/6 mice (loading of 20 µg total proteins in each well). Abbreviations are: normal  =  wild-type and GF  =  germ-free.

Furthermore, western blot analysis was carried out on plasma purified from wild-type or germ-free C57BL/6 mice to determine whether or not the presence of DING proteins in rodent was due to a symbiotic or contaminating microbe. In both wild-type and germ-free C57BL/6 plasma, a similar pattern was observed (**Figure 2**). This indicates that plasma DING proteins do not originate from bacteria but are directly produced by eukaryotic cells and are subsequently secreted in the circulating blood. Anti-DING polyclonal antibody revealed several bands ranging from 35 to 130 kDa, with a slightly different pattern from that observed in mouse tissues. As a case in point, 2 bands of HMW-DING at 130 kDa and 100 kDa and a shorter DING protein of 35 kDa were specifically observed. On the other hand, 2 bands corresponding to HMW-DING at 52 kDa and 71 kDa were shared with other mouse tissues. The band at 71 kDa is very strong, suggesting that this isoform is widely predominant in mouse plasma.

### DING Protein Detection in the Mouse Brain

**Figure 3:**
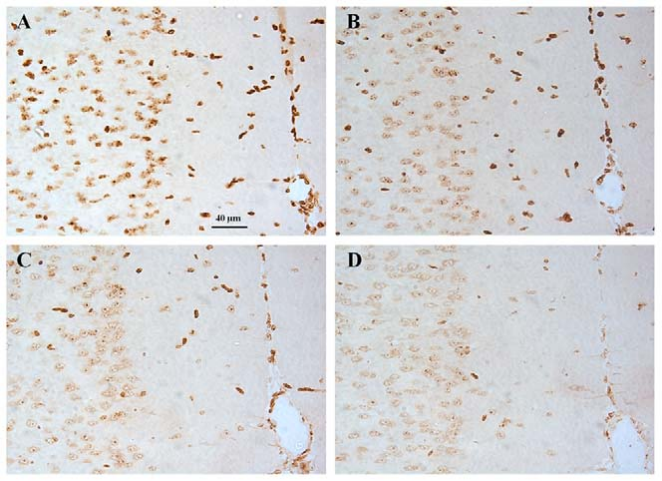
Competition assays to assess polyclonal antibody specificity. Anti-DING immunohistochemistry was performed on brain sections from control mice (panel A). In a competition experiment, 0.4 µg (panel B), 2 µg (panel C) or 10 µg (panel D) of human purified HPBP protein was added in the primary antibody incubation step. In each panel, the same brain area (cingulate cortex area) was photographed with the same magnification (see scale bar on panel A).

DING protein staining was detected in all cerebral regions of brain sections using polyclonal antibody. The specificity of the polyclonal antibody was verified in competition experiments where various quantities of purified HPBP (ranging from 0.4 to 10 µg per brain section) were added in the primary antibody incubation step (**Figure 3**). The addition of 0.4 µg HPBP (**Figure 3B**) is sufficient enough to induce a significant reduction of the intensity of DING immunolabeling previously observed in brain sections in absence of purified HPBP competitor (**Figure 3A**). An almost complete depletion of DING protein immunolabeling was obtained by the addition of either 2 or 10 µg of purified HPBP competitor (**Figures 3C and 3D**). This demonstrates the high specificity of polyclonal antibody raised against mouse DING proteins and HPBP.

**Figure 4:**
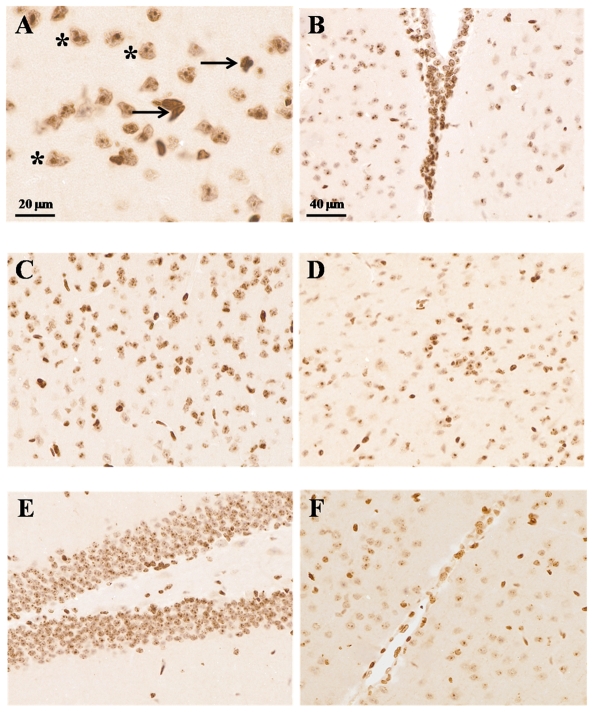
DING immunochemistry in the mouse brain. Different brain areas were photographed on control mouse brain sections labeled by anti-DING immunochemistry, using diaminobenzidine as revelator dye. The different brain regions are: the cerebral cortex (panels A and C), the sub-ventricular zone or SVZ (panel B), the thalamus (panel D), the granular layer of the dentate gyrus (panel E) and epithelial cells from blood vessels (panel F). Scale bars represent 20 µm for panel A or 40 µm for panels B to F. In panel A, black arrows indicate brain cells with intense DING labeling throughout the whole nucleus while black asterisks refer to brain cells with small patches of DING labeling in poorly labeled nuclei.

In the brain, DING labeling was exclusively detected in cell nuclei but following two different staining patterns. In the first one, the whole nucleus is strongly labeled (**Figure 4A**). This labeling can be either evenly distributed throughout the nucleus or some more intensely stained patches can be spotted in the labeled nuclei. In the second pattern, detected DING proteins appeared as small patches scattered in a poorly labeled nucleus (**Figure 4A**). The relative proportion of strongly- or poorly-labeled nuclei is dependent on the considered cerebral regions. For example, strongly-labeled nuclei are almost entirely present in epithelial cells constituting blood vessels (**Figure 4F**) and are dominant in cells located in the subventricular zone and the cerebral cortex (**Figures 4B and 4C**). In contrast, the majority of “poorly”-labeled nuclei are found in the lateral septum, the striatum and the hippocampus (dentate gyrus and CA1, CA2 or CA3 hippocampal fields). Regardless of the strongly- or poorly- stained status of nuclei, the distribution of DING protein-positive cells is also dependent on the related brain areas. In a semi-qualitative study, the highest number of labeled cells was observed in the granular layer of the dentate gyrus (**Figure 4E**), the CA1, CA2 and CA3 fields of the hippocampus and the subventricular zone (**Figure 4B**). To a lesser extent, the density of DING protein-labeled cells is also important in the hypothalamus, the corpus callosum, the amygdala, the choroid plexus and all cortices including cingular, piriform and cerebral cortices (**Figure 4C**). The quantity of DING protein-positive cells was low in the thalamus (**Figure 4D**), the lateral septum, the striatum and some hippocampal areas deprived of pyramidal neurons such as the stratum radiatum, the lacunosum moleculare layer and the molecular layer of the dentate gyrus.

Both H&P staining and DING protein immunochemistry were combined on same brain sections (co-labeling experiments) to ensure that all brain nuclei are positively labeled with anti-DING antibody (data not shown). In all checked cerebral regions, non-H&P-stained nuclei were deprived of DING protein labeling. Therefore, the density of DING protein labeling in a given brain area is directly correlated to the density of cell nuclei in the specific cerebral region.

**Figure 5:**
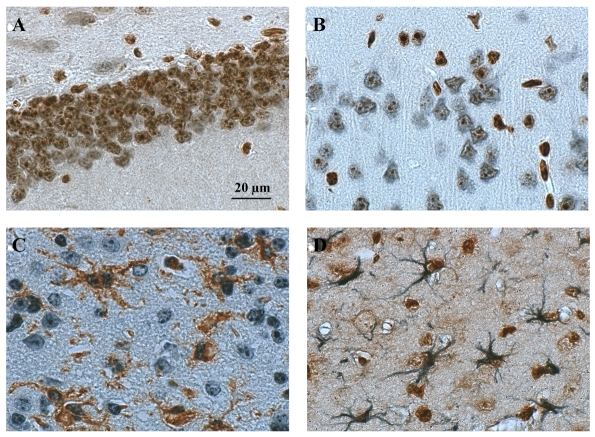
Characterization of DING-labeling in brain cells. Anti-DING/anti-NeuN double staining was performed in brain sections of control mice to detect DING-expressing neurons (panel A: granular layer of the dentate gyrus; panel B: cerebral cortex). Diaminobenzidine (brown labeling) and SG vector dye (blue labeling) were used to view DING and NeuN, respectively. Activated microglia (panel C) and astroglia (panel D) expressing DING proteins were revealed in brain sections of soman-poisoned mice with anti-DING/GSA lectin or anti-DING/anti-GFAP double staining immunochemistry, respectively. Blue staining was obtained with SG vector (labeling of DING proteins in microglia and GFAP in astrocytes) while brown staining was achieved using diaminobenzidine (labeling of DING proteins in astroglia and GSA lectin in microglia).

The presence of large numbers of DING protein-positive cells in the hippocampal CA1 field or in the granular layer of the dentate gyrus undoubtedly means that these positively-stained cells are neurons since these brain areas involve pyramidal neurons for the most part. As assessed by DING protein/NeuN double labeling (**Figures 5A and 5B**), co-localization of NeuN and DING proteins was observed in many cell nuclei confirming the presence of DING protein in neurons throughout the brain.

To address the issue as to whether DING protein was also present in astroglial or microglial cells, DING protein/GFAP or DING protein/lectin GSA double staining were performed in the brain of soman-poisoned mice, respectively. Usually, resting glial cells are not easy to detect due to the thinness of their perinuclear cytoplasmic processes and the smallness of their nuclei. Therefore, a mouse model containing activated glial cells with prominent cytoplasmic branching processes and hypertrophied nuclei, namely the soman-intoxicated mouse, was chosen. The presence of DING protein was demonstrated in numerous activated microglial or astroglial cells after soman poisoning, as shown in **Figures 5C and 5D**, respectively.

### DING Protein Detection in Other Mouse Tissues

**Figure 6:**
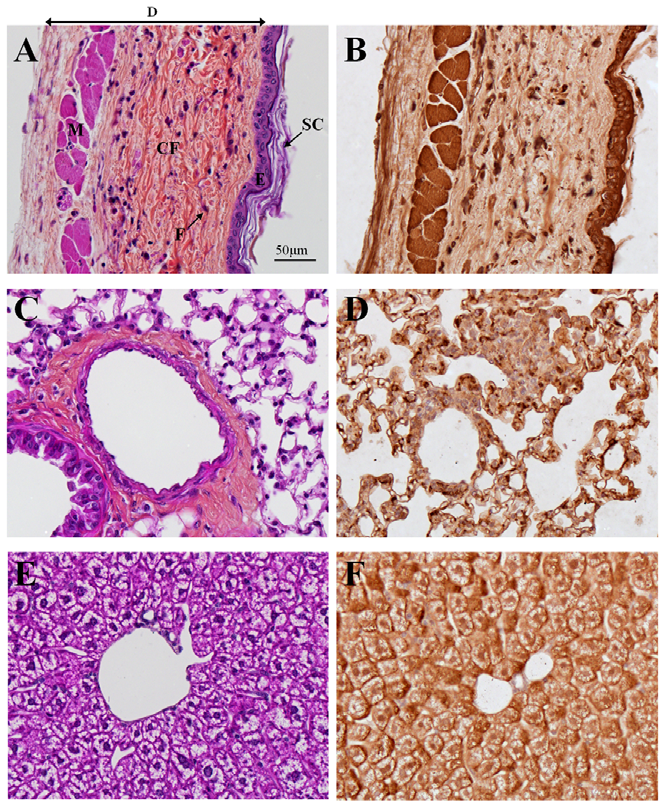
DING immunochemistry in different mouse tissues. Skin (panels A and B), lung (panels C and D) and liver (panels E and F) were collected from control mice and processed for hemalun-phloxin-saffron staining (panels A, C and E) or anti-HPBP immunochemistry (panels B, D and F). The same magnification was used for all the photographs (see scale bar on panel A). Abbreviations are: SC  =  *stratum corneum*; E  =  epidermis; D  =  dermis; F  =  fibroblast; CF  =  collagen fibers and M  =  cutaneous underlying muscles.

In the skin, a massive DING protein-positive staining was observed in the *stratum corneum*, epidermis (including basal keratinocytes) and cutaneous underlying muscles (**Figures 6A and 6B**). In the dermis, only fibroblasts were strongly labeled with anti-DING antibody. However, a weak DING protein labeling was found in the dermis extracellular matrix mainly consisting of collagen fibers (**Figures 6A and 6B**).

In the heart, muscle fibers were mildly stained with anti-DING antibody in their entirety, including cardiomyocyte nuclei. DING protein was also detected in the nuclei of endothelial cells from blood vessels (data not shown).

The presence of a heterogeneous DING protein labeling was observed in the aortic media and adventitia. An intense labeling was evidenced in the myocyte and fibrocyte nuclei while a slighter labeling was displayed in the aortic amorphous ground substance enriched in chondroitin sulfate. We observed a lack of DING protein staining in elastic concentric membranes and collagen fibrils of the aorta (data not shown).

In the lung, a positive DING protein labeling was identified in cells of the pulmonary alveoli and bronchioles with folded mucous membranes (**Figures 6C and 6D**). DING protein labeling is mainly concentrated in cell nuclei. A positive staining is also present in cytoplasm but in a more diffuse pattern.

In the liver, all the hepatocytes are stained with anti-DING antibody. A strong staining was localized in the nucleus and plasma membrane of hepatocytes while a weaker staining was noticed in the cytoplasm (**Figures 6E and 6F**).

Altogether, these results clearly substantiate the presence of DING protein in all tested mouse tissues. At the cellular level, the strongest labeling was generally localized in nuclei and to a lesser extent in the cytoplasm of considered cells. Moreover, it is noteworthy that DING proteins were rarely spotted in the extracellular matrix. Indeed, DING proteins were only found in the aortic amorphous ground substance or in the skin epidermis.

## Discussion

### Origin of Mice DING Proteins

The origin of DING proteins is prone to controversy [11]. Indeed, in the post-genomic era, their genes are still consistently missing from databases and the few partial nucleic sequences identified in non-annoted parts of genomes are highly similar to bacterial DING representatives, such as *Pseudomonas* DING proteins. In addition, their codon usage is similar to that of *Pseudomonas*, leading to the hypothesis of a bacterial origin [10]. Because of the numerous biological implications of DING proteins in eukaryotes, the hypothesis of *Pseudomonas* contamination has been discarded [11], [33], but the hypothesis of an unknown microbial symbiosis with eukaryotes remains a viable hypothesis [11]. In order to investigate the possibility of microbial symbiotic origin of DING proteins in eukaryotes, we analyzed wild-type and germ-free mouse plasma samples using western blot assays. The specificity of the used antibodies has been checked using competition assays (**Figure 3**). In addition, the experiment was performed using different kind of polyclonal antibodies, targeted against HPBP (**Figure 2**), and targeted against the very conserved N-terminal peptide of DING proteins (**supporting document – Figure S1**). The similar patterns obtained in both experiments evidence the specificity of antibodies against DING proteins. The DING protein profiles were compared in both wild-type and germ-free C56BL/6 mouse plasma. The present study shows a similar distribution of DING proteins in either wild-type or germ-free C57BL/6 mouse plasma. This indicates that the presence of DING proteins in eukaryotes is likely not to be related to microbial symbiosis nor to contaminations. Therefore, regarding data provided in this study, the more likely hypothesis concerning the origin of eukaryotic DING proteins is a synthesis by eukaryotic cells themselves.

In addition, the presence of mouse plasma DING protein is consistent with the recent purification of a human DING representative HPBP in the blood as published by Morales et al. [16]. The existence of DING proteins in mammalian plasma suggests therefore that these proteins are synthesized by eukaryotic cells and are subsequently secreted in the circulating blood. To date, the secretion route of DING proteins remains unidentified. The sequence of DING protein genes or the full sequencing of HMW-DING proteins is likely to shed light on the understanding of mechanisms involved in the secretion of these proteins.

### Omnipresence of DING Proteins in Mouse Tissues

As assessed by western blot analysis, DING proteins were detected in all explored mouse tissues, namely: brain, skin, heart, aorta, lung and liver. Our present results provide substantial evidence that the omnipresence of DING proteins in various mouse tissues is consistent with data previously published in other mammalian models. Indeed, the 40 kDa cotinine receptor, a member of the DING protein family, was purified from rat brains and sequenced [34]. More recently, the 38 kDa apolipoprotein HPBP has been isolated from human blood and its structure was solved using X-ray crystallography [16], [35]. HPBP being a apolipoprotein [16], it could be produced by hepatocytes and could be subsequently secreted in the circulating blood. Therefore, it is not surprising to detect DING proteins in both the mouse liver and plasma, in keeping with substantiated evidence from our experiments.

As mentioned above, only the 41 kDa DING protein was present in mouse liver, heart, brain, skin or lung. Further, the quantity of this protein was more or less similar in all investigated tissues. However, the distribution pattern of HMW-DING proteins was different depending on the nature of the related tissue. A total of four major HMW-DING at 140 kDa, 71 kDa (double bands), 62 kDa and 52 kDa were revealed in tissues, and two others at 130 kDa and 100 kDa were revealed in plasma samples. For example, the 52 kDa HMW-DING was predominant in the skin while the quantities of 140 and 71 kDa HMW-DING were more important in the liver as compared to the other tested mouse tissues. The diversity of HMW-DING proteins possibly results in different physiological involvements. The various forms of DING proteins detected in these assays, different than the classical 41 kDa form, might correspond to higher molecular weight DING protein precursors and truncated or maturated DING proteins. Because of the high sequence conservation within the DING protein family, these bands might also correspond to uncharacterized mouse DING proteins.

### Intracellular Localization of Mouse DING Proteins

DING proteins are mainly localized in the nucleus of every cell types present in all investigated mouse tissues. Generally, nuclear stainings are very intense, except in the brain where neuronal nuclei exhibit either strong or reduced labeling. However, as regards other cerebral cells such as microglia or astroglia, all nuclei are deeply stained with anti-DING antibody. Since we investigated the cellular localization of DING proteins with antibodies that recognizes various DING protein forms, the observed signal could be derived from any of these forms. The presence of DING proteins in cell nucleus is consistent with biological activities identified for some of these proteins. For example, bacterial PfluDING protein isolated from *Pseudomonas fluorescens* is competent to stimulate the proliferation of cultured human fibroblasts as assessed by ^3^H-thymidine incorporation experiment [7]. Another DING protein, p27^SJ^, suppresses expression of HIV-1 genome [36]. This suppression of expression is mediated by the physical and functional association of p27^SJ^ with human C/EBPβ transcription factor and viral Tat transactivator. C/EBPβ is a factor stimulating HIV-1 gene transcription and regulating the expression of several genes involved in mammalian cell proliferation or differentiation [3], [36], [37]. Tat transactivator is a viral protein triggering the transcription of HIV-1 viral genes. Moreover, p27SJ possess a phosphatase activity inducing a dysregulation at S and G2/M phases in cell cycles related to alteration of Erk1/2 phosphorylation state [38]. In addition, the X-DING-CD4^+^ seems to interact with transcription factors in the nucleus, and is believed to be involved in the resistance to HIV infection of non-progressive patients [22], [39]. Since DING proteins are clearly involved in cell cycle regulation and cell proliferation which are complex processes within the nucleus, it may implies a nuclear localization of these proteins for an optimal activity. Our present investigation provides substantial evidence of the nuclear localization of DING proteins.

The nuclear localization of DING proteins raises the issue of the pathways for these proteins to the nucleus after synthesis in the cytoplasm. The import of proteins into the nucleus is tightly regulated and specific nuclear localization signals (NLS) are required for the transport process [40], [41]. Concerning DING proteins, none of the available protein sequences indicate the presence of such a NLS. However, despite being mostly isolated, sequenced and characterized as 38–41 kDa proteins, DING proteins exist also as higher molecular weight proteins [4]. Our study provides evidence to that effect. Then, it could be assumed that NLS might be present in HMW-DING proteins rather than in mature DING proteins. The sequencing of these HMW-DING proteins would be useful to address this issue. DING proteins are also detected in the cytoplasm of liver, lung, skin and aortic cells. Nevertheless, the quantity of DING proteins in cytoplasm is much lower than the quantity revealed in nucleus. DING proteins are completely absent in the cytoplasm of cerebral cells, including neurons, microglia and astroglia. Moreover, the liver is the only tested tissue of our study for which DING proteins was massively localized in the plasma membrane, reinforcing the hypothesis of a possible secretion of these proteins from liver cells to the circulating blood, via a secretion of the DING protein through cell plasma membrane. Since our study demonstrates that 52 kDa and 71 kDa-DING proteins are both intracellular and secreted, this could argue in favor of an unconventional route for DING proteins secretion rather than a secretion pathway via N-terminal secretion signal peptides.

### Conclusion

Our study hereby, provides extensive data as regards intracellular DING protein localization in several mouse tissues. DING proteins seem to be ubiquitous in all tested tissues (i.e. brain, skin, heart, aorta, liver and lung) and exists as different isoforms, since we identified, in addition to the common 41 kDa-DING protein, High Molecular Weight DING proteins in all tested mice tissues. The localization of DING proteins is tissue-dependant: such proteins exhibit an intracellular localization in the brain while these proteins are detected either in the nucleus or cytoplasm of other investigated tissues. As for liver, DING proteins were also recovered in the plasma membrane, findings back up therefore the hypothesis of a possible secretion of DING proteins from liver to circulating blood, as evidenced by the presence of DING proteins both in intracellular and in plasma. Finally, the comparison of western blot profiles performed on blood plasma purified from wild-type and germ-free mice further evidenced the assumption that DING proteins are likely to be produced by eukaryotic cells.

## Supporting Information

Figure S1 Western Blot assays on mouse plasmas using anti-N-term antibodies.
(0.08 MB DOC)
