# Supplementary material for: Eukaryotic DING Proteins Are Endogenous: An Immunohistological Study in Mouse Tissues
Source: PLoS One. 2010 Feb 8;5(2):e9099. doi: 10.1371/journal.pone.0009099 (PMC2817009; doi:10.1371/journal.pone.0009099)
Supplement: Figure S1 — Western Blot assays on mouse plasmas using anti-N-term antibodies. (0.08 MB DOC) [file pone.0009099.s001.doc]

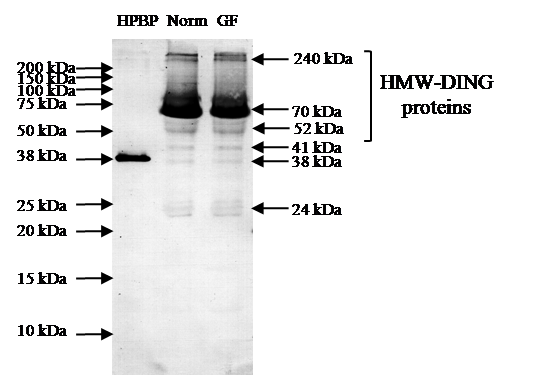


**Figure S1: Western blot analysis on plasma samples from normal and germ-free mouse using N-terminal targeted polyclonal antibodies**

Western blot was achieved with plasma from wild type and germ-free C57BL/6 mice (loading of 20 µg total proteins in each well). Abbreviations are: HPBP (Human Phosphate Binding Protein) as a reference, norm = wild-type and GF = germ-free.

**Methods:** The western blot analysis was performed as described previously in this study. For each tissue homogenate or plasma sample, 20 µg of total protein per well were loaded onto a 15% SDS-PAGE gel. The transfer was performed as described previously in the study. Membranes were incubated with 1:2000 polyclonal antibodies targeted against HPBP 20-residues N-terminal peptide (prepared by Genecust Company (Dudelange, Luxembourg)) and 1:2500 anti-rabbit antibodies (BioRad). The size of DING proteins bands was calculated as described previously in this study.

**Results and discussion:** The western blot analysis on plasma samples from normal and germ-free mouse using polyclonal antibodies targeted against the N-terminal peptide of HPBP reveals a similar pattern that the analysis performed with polyclonal antibodies targeted against HPBP (**figure 2**), albeit few differences. Bands are common to these two analysis, like the 52kDa and the very intense 70kDa bands. Higher MW bands can be observed, such as a 240kDa band. The differences in the observed bands patterns (presence of 24, 41 and 240 kDa) could stem from the presence and the conservation rate of the N-terminal peptide in the various DING proteins isoforms, that influence directly the affinity of N-terminal targeted antibodies.

The similarity between western blot analysis patterns using polyclonal antibodies anti-HPBP (**figure 2**) and using polyclonal antibodies targeted against the N-terminal peptide that is very conserved among the DING protein family (**supporting document – Figure S1**), constitute an evidence that polyclonal antibodies targeted against HPBP used in this study are specific to mouse DING proteins.
